# Considerations for the Maximum Heat Load and Its Influence on Temperature Variation of the Evaporator in Flat MHPs in Transient Regimes

**DOI:** 10.3390/mi13060979

**Published:** 2022-06-20

**Authors:** Ioan Mihai, Cornel Suciu, Claudiu Marian Picus

**Affiliations:** 1 Faculty of Mechanical Engineering, Automotive and Robotics, Stefan cel Mare University, 720229 Suceava, Romania; claudiu.picus@usm.ro; 2 Faculty of Electrical Engineering and Computer Science, Stefan cel Mare University, 720229 Suceava, Romania

**Keywords:** microchannels, flat MHP’s, heat load, evaporator, capillary radius

## Abstract

The present paper describes a series of considerations for the occurrence of capillary boundaries in flat micro heat pipes (flat MHPs) and the conditions required for their stable operation in relation to the working circumstances and to the type of liquids inside the pipes. Particularities of heat transfer in a flat MHP are analyzed for situations of either excessive or deficient working liquid. Depending on the physical properties of the working liquids (acetone, methanol and distilled water), the maximum rate of heat flow that can be applied to a flat MHP is determined analytically. The calculus is made with the assumption that constant vaporization of the liquid is ensured in the flat MHP’s evaporator, with no overheating. The considered analytical models allow for the evaluation of the liquid film thickness and the mass flow corresponding to the vaporization region. The temperature difference between the inner and outer walls of a flat MHP is found in the case of a transient regime and a variable thermal flow is applied in the evaporation region. The interior of flat MHPs was modeled in MATLAB using an FTCS (Forward-Time Central-Space) method, which is a finite difference method used for numerically solving the heat equation.

## 1. Introduction

Power electronic equipment is present in most modern specialties. The current extent of miniaturization requires cooling solutions capable of ensuring increased efficiency, reduced external power consumption, minimum dimensions and low-as-possible production costs [1,2]. Heat pipes and flat MHPs are among the systems able to ensure efficient cooling of electronic components, the latest ones are currently employed in many applications.

The first historical record of thermal pipes is in 1836 when Jacob Perkins used the principle of gravitational thermo-siphoning [3]. In 1929, F.W. Gay according to [3,4] used, for the first time, Perkins tubes to construct an air-to-air heat exchanger. It can be stated that modern heat pipes first appeared in 1944 when R.S. Gaugler (patent U.S. No. 2350348) [3,5] employed an internal structure that facilitated the motion of condensed liquid within a thermal tube. In 1962, Trefethen according to [6], proposed the use of thermal tubes in cosmic space equipment, and in 1964, Groover, et al., according to [7], were the first to introduce the expression “heat pipe”.

Micro Heat Pipes were first mentioned relatively late, in 1984, by T.P. Cotter [8]. These pipes are characterized by the fact that the mean curvature of the working liquid meniscus at the liquid-vapor interface is comparable in size to the inverse of the hydraulic radius of the flowing channel. Such devices were later introduced for semiconductor technology due to their extremely small dimensions [9].

Constructively, flat MHPs are divided into three regions namely, vaporization, adiabatic and condensation. The cyclic thermodynamic transformations of fluids inside a flat MHP are vaporization, due to absorbing heat from the power electronic component, vapor transport, through the adiabatic region, followed by condensation with the formation of liquid droplets. This step includes heat loss in the environment. The condensed liquid is then returned to the vaporization region by way of a “pumping” mechanism obtained via the capillarity effect in one or more internal capillary layers of flat MHPs.

Most cooling systems based on flat MHPs are passive devices, as they use the bi-phasic transformations of the working liquids. Even though flat MHPs are viable cooling solutions they present functional limits determined by their constructive shape, working liquid and the ability to transport important variable heat flows. Research carried out so far by other authors has focused on the cooling of flat MHPs, while Sprinceană and Mihai [2] propose an external evaporator cooling. The proposed cooling process takes place in addition to the cooling of the condenser with a fan and can be done by adding a cold liquid or using Peltier cells. The additional cooling of the evaporator leads in its turn, to an increase in the amount of liquid in the trapezoidal microchannels and thus of the capillary radius. Since the proposed idea is novel in the authors’ opinion, the present paper aims to determine by analytical calculations the maximum heat load in the case of additional evaporator cooling. In addition, since during operation, there is a significant change in the various rates of heat flow, another objective of the paper is to study the temperature variation in the evaporator wall under the considered conditions. For this purpose, the temperature difference between the inside and outside of the evaporator wall from the operation onset until the regime stabilization was calculated for a variation of the heat flow between 10 W and 40 W. The calculations were performed for the case of transient operating regimes in flat MHPs. The evaporator temperature change was also determined by numerical modeling of the heat equation using the FTCS finite difference method. To verify the idea of additional evaporator cooling, Sprinceană and Mihai {2] performed a series of experiments using various liquids inside flat MHPs such as distilled water, methanol and acetone. The results show that, regardless of the used liquid, external evaporator cooling strongly increases the maximum cooling capacity of the flat MHPs. A comparison of the experimental results obtained by the authors with analytical or numerical results including those of other researchers was made.

## 2. Factors That Influence the Maximum Heat Transport Capacity for a Flat MHP

**Figure 1:**
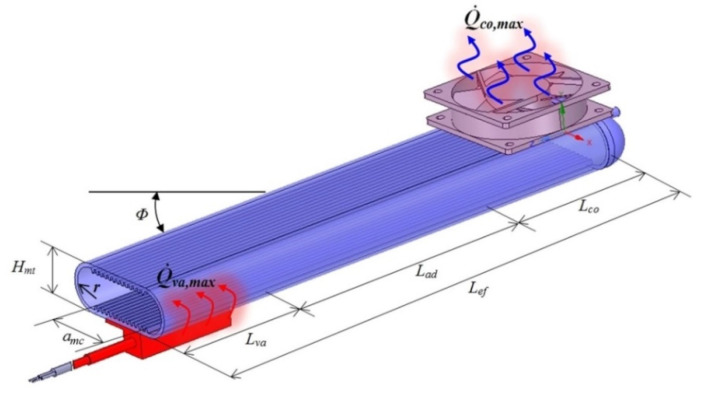
Operating areas and dimensional parameters of a flat MHP [2].

Figure 1 presents a flat MHP [2], where the operating regions can be observed, as well as some of the geometrical parameters.

The nomenclature from Figure 1 is: ${\dot{Q}}_{co,\max}$—maximum heat flow released at the condenser, *co*—condenser, *Φ*—angle between the flat MHP and the horizontal direction, *H_mt_*—external height of the flat MHP, ${\dot{Q}}_{va,\max}$—maximum heat flow applied at evaporator, *va*—evaporator; *r*—internal radius of the flat MHP’s body, *a_mc_*—useful width of rectangular microchannels, *L_va_*, *L_ad_*, *L_co_*—lengths of condensation, adiabatic and vaporization regions, respectively, *L_ef_*—effective length of the flat MHP’s working regions.

The internal capillary layers of a flat MHP ensure the motion of the condensed liquid phase due to their structure made of microchannels, micro-spheres made of synthesized copper and woven microfibers, etc. A series of hypotheses [3,10,11,12,13,14], were made in order to analyze the heat transport capacity of a flat MHP. It is assumed that flat MHP works when placed horizontally, and the wall is heated on one side by the thermal source (the power electronic component) via a conduction process.

Heat transfer between the inner capillary layer and the working liquid is accomplished only in a radial direction (the process in the axial direction is neglected). The working liquid and its vapor move only in an axial direction. The condenser of flat MHPs is not blocked by the liquid obtained through vapor condensation while the capillary layer is assumed isotropic and saturated with liquid. The change of the liquid’s meniscus in the microchannels of the capillary layer is determined by the capillary pressure.

The vapor and working liquid flow rates are considered constant and their flow laminar near the inner wall over the entire length of the flat MHP, and turbulent in the core of the vapor flow where the axial Reynolds number is greater than Re = 12,000 [15]. From a directional point of view, heat transfer from a flat MHP can either happen from exterior to interior (vaporization region) by receiving heat from the warm source in the vaporization region, inside the micro heat pipe (adiabatic area) when vapor moves from the vaporization region to the condensation region or from inside to the outside (condensation region). Heat transfer through the flat MHP’s microchannels can be conductive and convective, with and without changing of phase, depending on the work area.

In the vaporization region, generated vapor moves at high speed, “often several hundred mph and approaching the speed of sound” [16], from this region to the condensation region. In a stationary regime, at the liquid-vapor interface, the principle of momentum conservation must be obeyed at any point from both transverse and longitudinal cross-sections. The Laplace–Young equation can be applied to find the radius of the liquid meniscus formed at the liquid-vapor interface. The continuity equations for the liquid and vapor flows through trapezoidal microchannels of a flat MHP through the central section from the condenser to the evaporator and in reverse are presented in [11,17].

The flat MHP’s channels have been considered to have a trapezoidal cross-section. Starting from the relations given by [18,19] a code was written in Mathcad [2] and a series of parameters such as the equivalent hydraulic diameter, the Poiseuille number, the curvature radius of the liquid’s meniscus versus the angle of the meniscus with the wall of the trapezoidal microchannel (considered circular by hypothesis) were found. The same code allowed for finding the pressure variation and the loss of pressure for vapor in the evaporator region using relations from the models developed in [12,19,20,21].

The variation of capillary pressure on the axial direction of the trapezoidal microchannel was calculated by determining the values of the Reynolds number for the working fluids flow. The minimum condition required for undiminished heat transport capacity through a flat MHP [22,23,24] states that the interior maximum capillary pressure must be no less than the pressures of vapor and working liquid.

Improper functioning of a flat MHP is often associated with a phenomenon called capillary limit which is directly related to the volume of working liquid existent within the capillary layers. The capillary limit phenomenon can be correlated to the pressure and temperature regimes inside the flat MHP. This phenomenon consists of impeding the motion of the freshly condensed liquid in the condenser towards the vaporization region via mechanisms that will be later described. The capillary limit phenomenon may produce the “drying” of the vaporization region with negative effects upon the quantity of vapor produced and implicitly upon the maximum heat transport capacity.

***Case of excessive working liquid:*** When the quantity of liquid is in excess inside the flat MHP, the capillary limit may be attained. This is due to the fact that too much working liquid vaporizes and the volume of the generated vapor increases. As a result, the pressure inside the flat MHP rises.

Friction between the liquid and the walls of the microchannels generate adhesion forces in the boundary layer. The above-mentioned increase in internal pressure leads to augmented adhesion forces and implicitly to an alteration of the capillary limit (the pumping pressure through the capillary layer). As a consequence, the excess liquid formed in the condenser by vapor condensation cannot return to the evaporator by capillary effect at the same flow rate as in the regular operating regime. The flow rate decrease at the evaporator determines the diminishing or even cease of heat transfer, which means that the capillary limit has been reached.

***Case of insufficient working liquid:*** If a heat-generating thermal flux is applied to the evaporator zone and the working liquid is scarce inside the flat MHP, the whole quantity of liquid envisaged for cooling will vaporize in a short period of time. Reducing the quantity of vapor obtained in the evaporation zone will diminish the quantity of condensed fluid produced in the condenser of the flat MHP. This situation also leads to a failure to meet the condition of the capillary. The evaporator region will dry out and the flat MHP will cease to function properly.

For a correct estimation of the maximum heat transport capacity of a flat MHP the following parameters were taken into account: nature of the flow, Poiseuille, Reynolds and Nusselt numbers, liquid and vapor flow velocity in trapezoidal microchannels and sintered layers.

## 3. Evaluation of Maximum Heat Load through a Flat MHP

Flat MHPs are thermodynamic systems able to transport heat even for small temperature differences between the evaporator and condenser [25]. They are used to transfer and transform the rate of heat flow [26]. This fact is a feature related to the bi-phase transformations and internal pressure. The inner structure of a flat MHP varies from one case to another but some of the elements are found in all constructive types. Further, the transport capacity is linked to the effective heat flux.

### 3.1. Analytical Determination of the Maximum Heat Flux Passing through the Evaporator of a Flat MHP

According to [3,27], the Young–Laplace conditions concerning the total variation of capillary pressure must be fulfilled so that the condensed liquid from the condenser returns to the evaporator through the internal capillary structure of a flat MHP:

(1) $$\Delta P_{cp,t}\geq \Delta P_{l}+\Delta P_{v}+\Delta P_{g},$$

where Δ*P_cp,t_*—total drop of capillary pumping pressure, Δ*P_l_*—liquid pressure drop in microchannels, Δ*P_v_*—vapor pressure drop along the flat MHP, Δ*P_g_*—pressure drop due to gravity (only for the flat MHP working tilted with respect to the horizontal direction).

**Figure 2:**
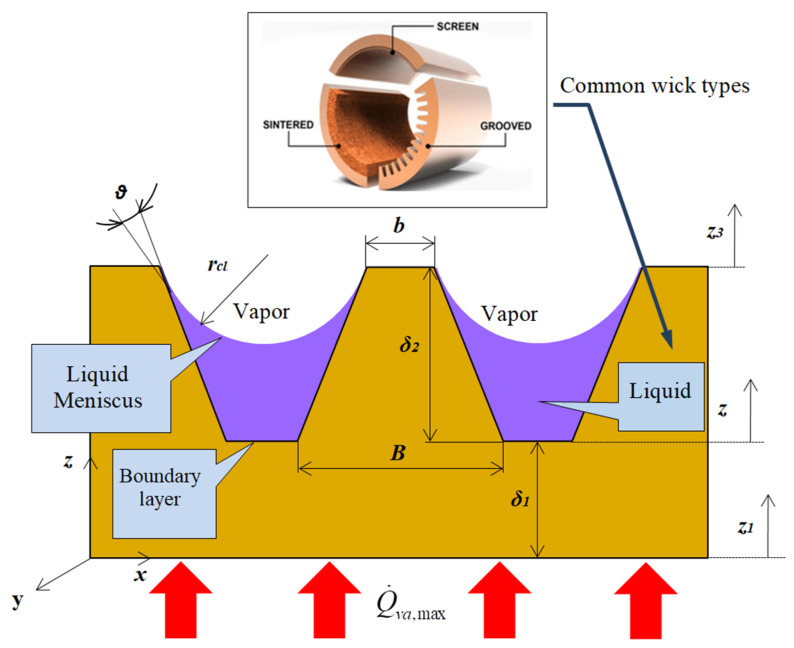
Cross-section through a flat MHP with capillary layer made of trapezoidal microchannels.

The geometric parameters of a flat MHP over the cross-section of the trapezoidal microchannels filled with working liquid and details for the common liquid-wick region [28], are shown in Figure 2.

The notations from Figure 2 are presented subsequently when used in equations. For nominal loads, Boughey B.W. [29] associated the capillary limit to the evaluation of thermal limit for which proper operation of flat MHP is ensured. As inside the flat MHP different working liquids are used, the surface tension of the liquid *σ_l_*, is considered and the following inequality is written:

(2) $$\Delta P_{cp,t}\geq \;\;\frac{2\sigma_{l}}{r_{cl}}.$$

The pressure drop of the liquid flowing inside the flat MHP [29], is:

(3) $$\Delta P_{l}=\frac{\mu_{l}{\dot{m}}_{l}L_{e}}{\rho_{l}\left(\sum_{i=1}^{N_{mc}}A_{mc},i\right)k_{l}},$$

where $\mu_{l}$—dynamic viscosity of the liquid, ${\dot{m}}_{l}$—mass flow of the liquid, *L_e_*—length of the evaporator (see Figure 1), $\rho_{l}$—liquid density, $\sum_{i=1}^{N_{mc}}A_{mc},i$—the sum of areas for *N_mc_* number of microchannels where the liquid flows, $k_{l}$—the effective thermal conductivity of the liquid-wick region. 

The pressure drop of vapor passing inside the flat MHP from the evaporator to the condenser is given by:

(4) $$\Delta P_{v}=\frac{\mu_{v}{\dot{m}}_{v}L_{va}}{\left(a_{mc}H_{mt}+\pi r^{2}\right)\rho_{v}k_{v}},$$

where $\mu_{v}$—dynamic viscosity of vapor, ${\dot{m}}_{v}$—mass flow of vapor, *L_va_*—length of the evaporator, *a_mc_*—width of the region with microchannels (see Figure 1), *H_mt_*—interior height of the flat MHP, *r*—interior radius of the flat MHP, *ρ_v_*—vapor density, *k_v_*—effective thermal conductivity of the vapor-wick combination.

The liquid pressure drop due to gravitational acceleration is:

(5) $$\Delta P_{g}=\rho_{l}L_{ef}g\sin\Phi ,$$

where $\rho_{l}$—density of the liquid from microchannels, *L_ef_*—effective length of the flat MHP, *g*—the gravitational acceleration, Φ—angle made by the flat MHP with the horizontal direction (see Figure 1). The pressure drop of the liquid Δ*P_g_* due to gravity can be zero, positive or negative, depending on the values of sinΦ. The total area of microchannels is considered in calculus. When the inequality from relation (1) is fulfilled, it can be said that the capillary limit is influenced by the flow of the liquid from the condenser toward the evaporator and by the pressure drops of both liquid and its vapor.

When the heat flux is applied only on one side of the vaporization region of a flat MHP [20,29,30], the produced capillary limit differs from when it is applied on both sides. Theoretically, in the case of heat flux being applied on both sides of the evaporator the capillary limit is superior to the case of one side application. For the situation of a flat MHP placed horizontally where the heat flux is applied on one side, only the microchannels from that side are considered for computation.

Functionally, the flat MHP is in a state of thermal equilibrium and thus the mass flow of liquid vapor and that of liquid from the evaporator are equal in the flow cross-section. The equation of energy balance is obtained taking into account [29,31,32] ${\dot{Q}}_{va,\max}$—the maxim heat flux ($\dot{Q}=\frac{\partial Q}{\partial t}$ quantity of heat, *Q*, transmitted over time (*t*)) that can be transported from the evaporator zone (Figure 1), ${\dot{m}}_{l}$—mass flow rate of the liquid, ${\dot{m}}_{v}$—mass flow of vapor through the flow cross-section in the evaporator’s elements and *h_lg_*—latent heat at constant pressure:

(6) $${\dot{m}}_{l}=-{\dot{m}}_{v}=\frac{{\dot{Q}}_{va,\max}}{h_{lg}}\;.\;\;\;$$

Neglecting the pressure loss due to vaporization and condensation, in 1995, Faghri [29], deduced the equation for the capillary limit (*Q_cpl_*_,max_), given by:

(7) $$Q_{cpl.\max}=\frac{\Delta P_{cp,t}-\Delta P_{g}}{f_{l}-f_{v}},$$

where *f_l_* and *f_v_* are the coefficients of friction losses for liquid and vapor, respectively. For the case of heat flux applied on one side of the flat MHP, the total maximum drop of capillary pumping pressure is:

(8) $$\Delta P_{cp,\max}=\frac{12\mu_{v}{\dot{m}}_{v}{\bar{L}}_{ef}}{\rho_{v}\left(aH_{mt}+\pi r^{2}\right)\delta_{v}^{2}}+\frac{12\mu_{l}{\dot{m}}_{l}{\bar{L}}_{ef}}{\rho_{l}\sum_{i=1}^{N_{mc}}A_{mc}\;\Psi },$$

where *δ_v_* is the mean thickness of vapor core in the cross-section of the flat MHP and *ψ* represents the permeability of the capillary layer. The mean length of the flat MHP is assumed ${\bar{L}}_{ef}=\frac{L_{va}+L_{co}}{2}$ since the liquid and the vapor pass over a shorter track than the actual one. The balance equation can be written as:

(9) $$\frac{2\sigma_{l}}{r_{cl}}={\bar{L}}_{ef}\frac{{\dot{Q}}_{va,\max}}{h_{lg}}\left(\frac{12\mu_{v}}{\rho_{v}\left(aH_{mt}+\pi r^{2}\right)\delta_{v}^{2}}+\frac{\mu_{l}}{\rho_{l}\sum_{i=1}^{N_{mc}}A_{mc}\;\psi }\right),$$

where *r_cl_* is the liquid meniscus radius in the channels of the flat MHP and *h_lg_* is the latent heat of vaporization for the working liquid. The maximum rate of heat flux to be transported from the evaporator region to the condenser is obtained as:

(10) $${\dot{Q}}_{va,\max}=\frac{2\sigma_{l}h_{lg}}{r_{cl}{\bar{L}}_{ef}\left[\frac{12\mu_{v}}{\rho_{v}\left(aH_{mt}+\pi r^{2}\right)\delta_{v}^{2}}+\frac{\mu_{l}}{\rho_{l}\sum_{i=1}^{N_{mc}}A_{mc}\;\psi }\right]}.$$

### 3.2. Evaluation of Maximum Heat Flux of the Evaporator of a Flat MHP in Relation to the Capillary Radius and the Working Liquids

The calculations were made using a Mathcad environment [33] and various calculus codes were developed by Mihai and Sprinceana [2]. For the trapezoidal microchannels of the flat MHP the next geometrical parameters were considered (Figure 2): length of trapezoidal solid small base *b =* 0.200 × 10^−3^ m, length of the large base *B =* 0.270 × 10^−3^ m and the height *δ*_1_ *=* 0.275 × 10^−3^ m.

The capillary radius was found via calculations for different values of the angle *θ* and it was established that it can take values in the range *r_cpl_ =* 3.48 × 10^−4^÷9.00 × 10^−4^ m. The heat exchange takes place through a total surface of the trapezoidal microchannels *A_mc_*= 9.454 × 10^−4^ m^2^ for a length of the evaporator *L_va_ =* 30 × 10^−3^ m, of the adiabatic zone *L_ad_* = 80 × 10^−3^ m and *L_co_ =* 40 × 10^−3^ m of the condenser, respectively. The total length of the flat MHP is *L_ef_ = L_va_ + L_adb_ + L_co_*. The thermo-physical properties of the working liquids typically used in flat MHP [34,35,36,37], are presented in Table 1.

**Figure 3:**
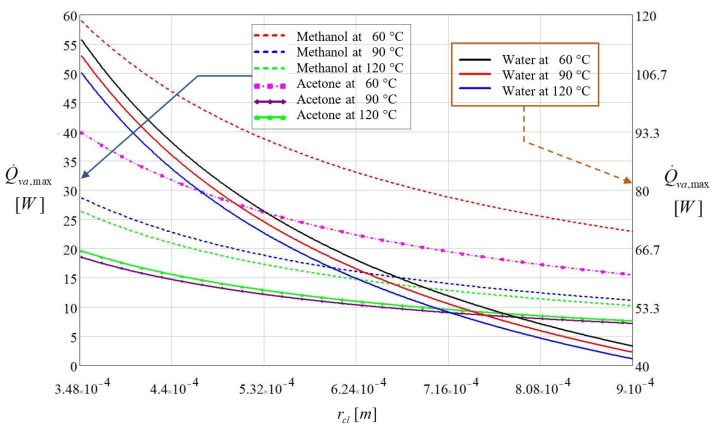
Maximum heat flux rate as a function of capillary radius and temperature for various fluids.

Starting from the parameters illustrated in Table 1, the maximum heat flux ${\dot{Q}}_{va,\max}$ [2], was calculated as a function of the capillary radius of the liquid meniscus from the trapezoidal microchannel, and its evolution is represented in Figure 3. In order to analyze the maximum heat flux rate that can be transported in the evaporator region of a flat MHP when the capillary limit is reached, the latent heat of vaporization *h_lg_* dependent on the working fluid was considered in the calculus.

Examining the results illustrated in Figure 3 it can be observed that distilled water has the greatest heat transport capacity from the evaporator to the condenser. The maximum heat flux transported from the vaporization region of the flat MHP increases as the capillary radius r_cpl_ diminishes.

For the distilled water, calculus yielded a maximum heat flux ${\dot{Q}}_{va,\max}=114.37W$ at *T =* 60 °C corresponding to a capillary radius *r_cl_ =* 3.48 × 10^−4^ m. When the vaporization region is overheated at a temperature *T =* 120 °C, the calculus is conducted to a heat flux value of ${\dot{Q}}_{va,\max}=106.62W$ for the same value of capillary radius.

Methanol and acetone show completely different behavior. Considering the same capillary radius as for the distilled water, the maximum heat flux corresponding to a temperature of 60 °C is 60.24 W for methanol and 21.27 W for acetone. Increasing the temperature to 120 °C, the resulting heat flux was 44.43 W for methanol and 19.12 W for acetone. Methanol and acetone are liquids with vaporization temperatures in the vicinity of 60 °C and for these cases, the variation of the maximum rate of heat flow is seriously influenced by the latent heat of vaporization.

The plots shown in Figure 3 reveal that the heat flux applied in the vaporization region influences the quantity of produced vapors. It depends on the nature of the working fluid and on the value of the vaporization temperature. For too high rates of vaporization, the quantity of produced vapors exceeds the flow rate of the condensed vapors produced in the condenser of a flat MHP. In this case, a dry region appears inside the evaporator. For the three analyzed liquids, the maximum rate of heat flow to be transported by a flat MHP reduces with the increase in the capillary radius and increase in temperature.

## 4. Temperature Variation during Transient Regime for a Flat MHP

The constructive parameters of the flat MHP presented in Figure 1 and Figure 2 are used for calculations. It is assumed that the flat MHP has a base made of copper and a liquid-wick structure accomplished by longitudinal microchannels with a trapezoidal cross-section.

### 4.1. Temperature Variation during Transient Regime through the Wall and Liquid-Wick Structure for the Evaporator of the Flat MHP

The equation of the thermal conduction for the non-stationary regime, unidirectional in the wall of the flat MHP that has not included the wick structure [1,13,38,39], is:

(11) $$\rho_{Cu}c_{p,Cu}\frac{\partial T_{p,va}}{\partial t}=k_{Cu}\frac{\partial^{2}T_{p,va}}{\partial z_{1}^{2}},\;\;\;for\;\;0\leq \delta_{tot}\leq \delta_{1},\;\;\;\;t\geq 0,\;\;\;\delta_{tot}=\delta_{1}+\delta_{2}.$$

where *ρ_Cu_*—copper density, *c_p,Cu_*—specific heat of copper, *T_p,va_*—temperature of the evaporator, *k_Cu_*—thermal conductivity of copper, *t*—time, *z*—radial direction (Figure 2).

For the microchannels composing the internal capillary layer (*mc* index) the Equation (11) can be written as:

(12) $${\left(\rho \;c_{p}\right)}_{eq}\frac{\partial T_{mc}}{\partial t}=k_{eq}\frac{\partial^{2}T_{mc}}{\partial z_{2}^{2}},\;\;\;for\;\;\delta_{1}\leq \delta_{tot}\leq \left(\delta_{1}+\delta_{2}\right),\;\;\;\;t\geq 0,$$

where the index “*eq*” has the meaning of “equivalent term”. The value of the temperature on the exterior side of the wall of the flat MHP (in the central zone of the evaporator) is considered equal to the external temperature of the heat source associated with an electronic component. The heat transfer in the vaporization region of the flat MHP is non-stationary, unidirectional along *z* axis (Figure 2); it is conductive in the region of *δ* thickness, mixed regime in the *δ*_1_ region (conductive in the solid sector of the trapezoidal microchannels and convective in the occupying liquid) and convective along *z*_3_ direction that corresponds to the motion of the vapors inside the flat MHP. For a non-stationary regime and considering the temperature generated at the wall by the heat source, the equations for the temperature variation in the wall of the evaporator and of the microchannel of the flat MHP can be written as:

(13) $$\Theta_{p,va}\left(t,z_{1}\right)=T_{p,va}\left(t,z_{1}\right)-T_{i},$$

(14) $$\Theta_{mc}\left(t,z_{2}\right)=T_{mc}\left(t,z_{2}\right)-T_{i},$$

where Θ*_p,va_* (*t*,*z*_1_), Θ*_mc_* (*t*,*z*_2_)—the difference in temperature between the wall of the evaporator and the one from inside the microchannel, *t*—time, *z*_1_—the thickness of the wall, *z*_2_ thickness of the liquid-wick structure which in this particular case corresponds to the microchannels filled with working liquid (Figure 2), *T*_*p*,*va*_ (*t*,*z*_1_) temperature of the evaporator’s wall, identical to the one of the warm source, *T*_*p*,*va*_ (*t*,*z*_2_)—temperature at the tip of the microchannel’s nervure, *T*_*i*_—initial temperature.

At the moment t=0, depending on the considered thickness of the wall (δ=0) up to the base of the trapezoidal microchannels of the flat MHP, the Equations (13) and (14) become:

(15) $$t=0|\begin{array}{c} \Theta_{p,va}\left(t,z_{1}\right)=0\;\;\;\Rightarrow \;T_{p,va}\left(t,z_{1}\right)-T_{i}=\;0\\ \Theta_{mc}\left(t,z_{2}\right)=0\;\Rightarrow T_{mc}\left(t,z_{2}\right)-T_{i}=0\;\;\;\;\; \end{array}\;.$$

It is assumed that the general equation for the distribution of temperature through the wall of the flat MHP in the evaporation zone has the form of a second-degree polynomial with respect to *z*:

(16) $$\Theta_{p,va}\left(t,z_{1}\right)=a\left(t\right)+b\left(t\right)z_{1}+c\left(t\right)z_{1}{}^{2}.$$

When a thermal flow (generated by a source of heat) is applied on the external surface of the wall of the flat MHP the wall of the evaporator will warm up through conductive thermal transfer. After traversing the wall of the flat MHP the rate of heat flow will worm up the working liquid from the trapezoidal microchannels ensuring the capillarity. By hypothesis, at the moment *t =* 0 there is no variation of temperature at the inner and outer sides of the wall of the condenser of the flat MHP. It is considered the instantaneous distance for the displacement of the lines of the heat field through the wall of the flat MHP, *δ_T_*, and ${\dot{q}}_{va}$ the density of thermal flow (internal source of generating heat) in the evaporation zone. The boundary conditions for the wall of the evaporator are stated:

(17) $$\left\{ \begin{array}{l} z_{1}=0\;\;\;\;\;\;\;\;\;\;\;\;\;\;\;\;\;\;\;{\dot{q}}_{p,va}=-k_{Cu}\frac{\partial \Theta_{p,va}\left(t,z_{1}\right)}{\partial z_{1}}\\ z_{1}={\delta_{T}\left(t\right)|}_{\max}\;\;\;\;\;\;\;\;\frac{\partial \Theta_{p,va}\left(t,z_{1}\right)}{\partial z_{1}}=0 \end{array}\right..$$

Considering the temperature variation for transient regime, Equation (12) becomes:

(18) $$\rho_{Cu}c_{p,Cu}\frac{\partial \Theta_{p,va}}{\partial t}=k_{Cu}\frac{\partial^{2}\Theta_{p,va}\left(t,\delta \right)}{\partial z_{1}{}^{2}}.$$

Using the boundary conditions from Equation (17) and the Equation (12), it becomes:

(19) $$a\left(t\right)=\frac{{\dot{q}}_{p,va}\delta_{T}\left(t\right)}{2k_{Cu}},{}^{}b\left(t\right)=-\frac{{\dot{q}}_{p,va}}{k_{Cu}},{}^{}c\left(t\right)=\frac{{\dot{q}}_{p,va}}{2k_{Cu}\delta_{T}\left(t\right)}.$$

The temperature variation in the wall of the evaporator with respect to time and to the thickness of the wall of the flat MHP, when the rate of heat flow passes through it is:

(20) $$\Theta_{p,va}\left(t,z_{1}\right)=\frac{{\dot{q}}_{p,va}\delta_{T}\left(t\right)}{2k_{Cu}}{\left(1-\frac{z_{1}}{\delta_{T}\left(t\right)}\right)}^{2}.$$

Since for plane-parallel walls both the density of the rate of heat flow ${\dot{q}}_{p,va}$ and the rate of heat flow ${\dot{Q}}_{va}$ are conserved, the single unknown from Equation (20) is *δ_T_(t)*. The rate of heat flow that passes the solid region (without microchannels) of the flat MHP between *z*_1_ and *z*_2_ is:

(21) $${\dot{Q}}_{va}={\dot{q}}_{p,va}\;A_{p,va}.$$

The notations from Equation (21) are: ${\dot{Q}}_{va}$—the thermal flow that moves inside the evaporator, *A_p,va_*—the surface of the evaporator’s wall that is crossed by the thermal flux. It must be considered that heat exchange is a transient process. As the material of the wall of the flat MHP accumulates heat, the coefficient of thermal diffusivity must be considered. The equation of the thermal conduction is integrated in order to calculate the evolution in time of the distance of the thermal field pattern *δ_T_(t)*, with respect to the wall:

(22) $$\int_{0}^{\delta_{T}\left(t\right)}\frac{\partial \Theta_{p,va}\left(t,z_{1}\right)}{\partial t}dz_{1}=\int_{0}^{\delta_{T}\left(t\right)}a_{p,va}\frac{\partial^{2}\Theta_{p,va}\left(t,z_{1}\right)}{\partial z_{1}{}^{2}}dz_{1}\;,$$

where:

(23) $$a_{p,va}=\frac{k_{Cu}}{\rho_{Cu}c_{p,Cu}},$$

is the coefficient of thermal diffusivity of the evaporator’s wall. The difference in temperature Θ_*p*,*va*_ (*t*,*z*_1_) from Equation (20) is replaced into the relation (22) and after solving, it results:

(24) $$d\delta_{T}^{2}\left(t\right)=6a_{p,va}dt.$$

By integrating the Equation (24), the distance traveled by the lines of the thermal field through the wall of the evaporator as a function of the material thermal diffusivity coefficient and of time is obtained:

(25) $$\delta_{T}\left(t\right)=\sqrt{6a_{p,va}t}.$$

**Figure 4:**
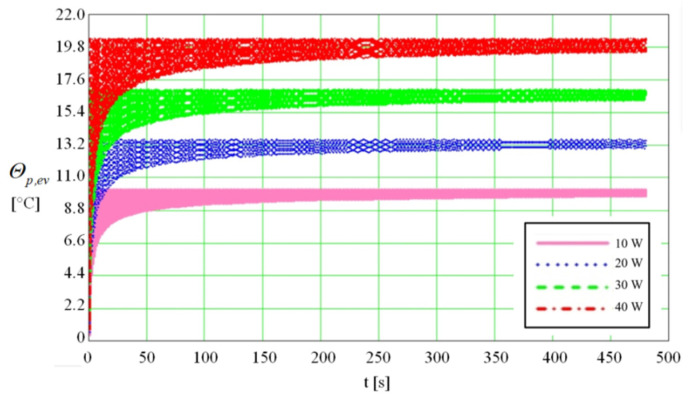
Difference in temperature versus time in the wall of the evaporator of flat MHP for different values of the thermal flow (10 ÷ 40 W).

The surface of the evaporator where the thermal flow is applied was found A_va_ = 9.88 × 10^−4^ m^2^ for a thickness of the evaporator’s wall, *δ*_1_. The variation of temperature difference as a function of time (480 s) in the wall of the evaporator of the flat MHP (from the side of the wall in contact with the source of heat to the distance *δ*_1_ (Figure 2) is represented in Figure 4. The outer side of the flat MHP is subjected to a variable thermal flow in the range of 10 ÷ 40 W. The calculus was made until the stabilization of the thermal regime.

From the plot presented in Figure 4, it is observed that the difference in temperature in the wall of the flat MHP is higher as the rate of heat flow increases. The thermal balance is reached when after a certain time the difference in temperature between the two faces of the wall of the flat MHP becomes constant. From the calculus it results that the period necessary to attain the thermal balance differs as a function of the values of the input rate of heat flow. For an input rate of heat flow of 10 W the temperature stabilizes after 148 s while for the maximum value of 40 W the time required for attaining the steady temperature increases to 396 s.

**Figure 5:**
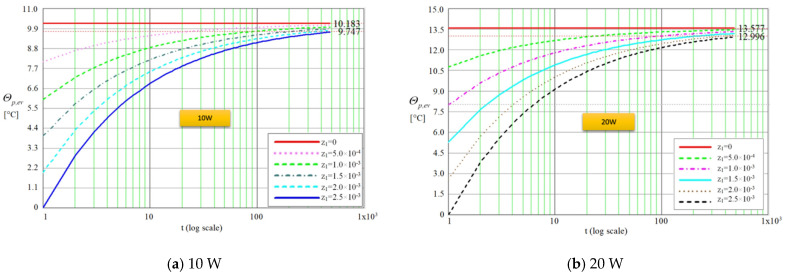

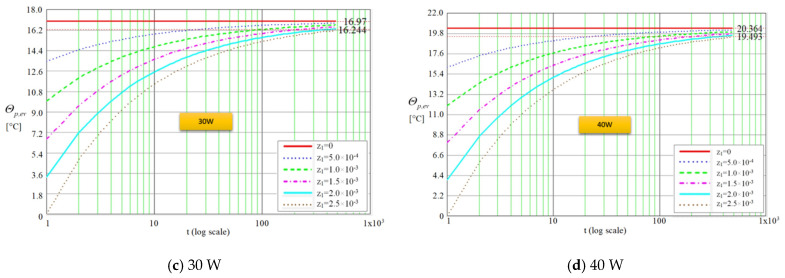
Difference in temperature versus time through the flat MHP wall for various rates of heat flow.

Using the same code, for the same time duration of 480 s (on logarithmic scale), the difference in temperature between the outer and inner faces of the wall of the evaporator was obtained for rate of heat flow values of 10, 20, 30 and 40 W and for wall thickness in the range 0÷2.50 × 10^−3^ m, were traced as shown in Figure 5.

From the plots presented in Figure 5a–d it is observed that the entire thickness of the wall of the evaporator is passed by the lines of the thermal field during a period of time until *480 s*. It can be noticed that after the crossing of the thickness *δ*_1_, (Figure 2), the temperature difference reaches a maximum of 10.18 °C for 10 W and 20.36 °C at 40 W, respectively. After passing the copper layer of thickness *δ*_1_, the rate of heat flow will pass through the liquid-wick structure of thickness *δ*_2_ (Figure 2). Taking into account that z_2_ represents the heat propagation direction, the temperature variation in the liquid-wick structure can take the form:

(26) $$T_{mc}\left(t,\delta_{2}\right)=a_{1}\left(t\right)+b_{1}\left(t\right)z_{2}+c_{1}\left(t\right)z_{2}{}^{2}.$$

The boundary conditions for the conductive heat transfer for unidirectional transient regime through the fins of the microchannels are:

(27) $$\left\{ \begin{array}{l} z_{2}=0\;\;\;\;\;\;\;\;\;\;\;\;\;\;\;{\dot{q}}_{mc}\;=-k_{eq}\frac{\partial T_{mc}\left(t,z_{2}\right)}{\partial z_{2}}\\ z_{2}=\;{\delta_{2,T}\left(t\right)|}_{\max}\;\;\;\;\frac{\partial T_{mc}\left(t,z_{2}\right)}{\partial z_{2}}=0 \end{array}\right.,$$

where *δ*_2,*T*_*(t)*—is the distance passed by the rate of heat flow through the trapezoidal microchannels (with the maximum value *δ*_2_), *k_eq_*—is the equivalent coefficient of thermal conduction for the liquid-wick structure. It is considered that the wall has a homogenous and isotropic structure. Analogous to the precedent case, the energy balance for the evolution of rate of heat flow through the liquid-wick structure is written as:

(28) $$T_{mc}\left(t,z_{2}\right)=T_{p,va}\left(t\right){\left[1-\frac{z_{2}}{\delta_{2,T}\left(t\right)}\right]}^{2}\;\;\;;\;\;\;{\dot{q}}_{mc}=\frac{2k_{eq}T_{mc}\left(t,z_{2}\right)}{\delta_{2,T}\left(t\right)}.$$

The Fourier equation, for passing through the liquid-wick structure of the flat MHP with *δ*_2_ thickness, for unidirectional (along *z*_2_ direction) transient regime is:

(29) $$\frac{\partial T_{mc}}{\partial t}=\frac{k_{eq}}{\rho_{eq}c_{p}{,}_{eq}}\frac{\partial^{2}T_{mc}}{\partial z_{2}{}^{2}}+\frac{{\dot{q}}_{va}}{\rho_{eq}c_{p,eq}}.$$

The energy balance for the capillary layer made of trapezoidal microchannels is:

(30) $$\int_{0}^{\delta_{2,T}\left(t\right)}\frac{\partial T_{mc}\left(t,z_{2}\right)}{\partial t}dz_{2}=\frac{\delta_{2,T}\left(t\right)}{2k_{eq}T_{mc}\left(t,z_{2}\right)}\;\int_{0}^{\delta_{2,T}\left(t\right)}a_{mc}\frac{\partial^{2}T_{mc}\left(t,z_{2}\right)}{\partial z_{2}{}^{2}}dz_{2},$$

where *a_mc_* is the coefficient of thermal diffusivity of the microchannel.

(31) $$T_{mc}\left(t,z_{2}\right)=\frac{{\dot{q}}_{va}{\left(6\frac{k_{eq}}{\rho c_{p,eq}}t\right)}^{1/2}}{2k_{eq}}\;{\left[1-\frac{z_{2}}{\delta_{2,T}\left(t\right)}\right]}^{2}\;.$$

**Figure 6:**
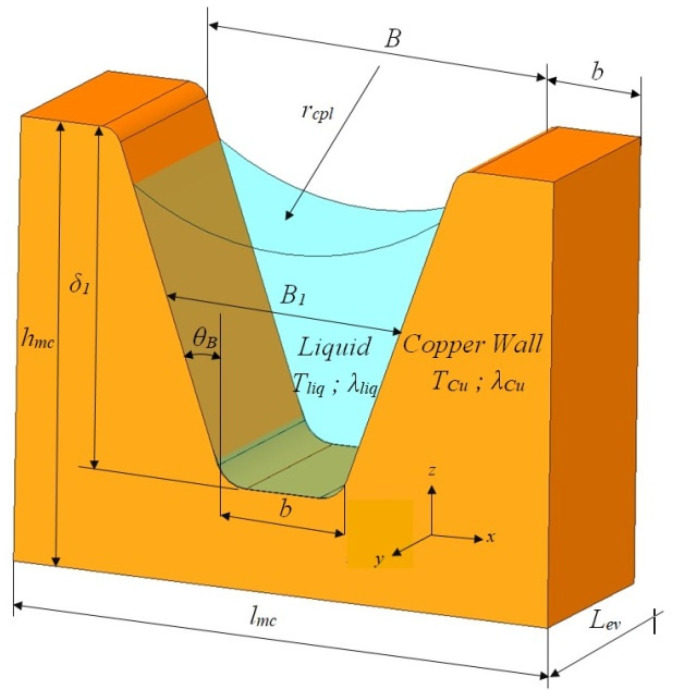
Geometry of the cross-section through a trapezoidal microchannel of the flat MHP [2].

In order to find the equivalent thermal conductivity of the evaporation region, there are considered the materials of the wall and of the capillary layer of the flat MHP together with the working liquid. These all together will have an equivalent thermal conductivity. According to Figure 6, it is considered that the trapezoidal microchannel is totally or partially filled with liquid.

In the evaporation zone, the microchannel is not totally filled with liquid. This fact is explained by the permanent vaporization of the working liquid along the entire path between the condenser and evaporator. Conversely, in the condenser’s region, due to permanent condensation of the vapors, the liquid may exceed the height of the microchannels. For the working fluid in the evaporator microchannels, a longitudinal section of a right-angled trapezium is formed between the condenser and the evaporator.

The trapezoidal microchannels are built into the copper wall, on the inside of the flat MHP. As the thickness of the liquid layer and the velocity of the liquid through the microchannels of the evaporator decrease, the convective phenomenon may be neglected. The hypotheses considered for calculus are that the heat transfer through the liquid-wick structure is conductive and under the same conditions for each fin of the microchannel, while for the convective heat transfer through the liquid from the microchannels, the dimensions of the microchannels are considered. For the vaporization case at the liquid-vapors interface [40], the coefficient of convective thermal transfer is estimated based on the kinetic theory of gases:

(32) $$h=\frac{2\kappa_{a}}{2-\kappa_{a}}\frac{\rho_{v}h_{\mathrm{lg}}^{2}}{T_{v}}{\left(2\pi \frac{R}{M}T_{v}\right)}^{-1/2}\left(1-\frac{P_{v}}{2\rho_{v}h_{\mathrm{lg}}}\right)\;,$$

where: *κ_a_*—thermal accommodation coefficient, *ρ_v_*—density of vapors, *h*_lg_—latent heat of vaporization of the vapors, *T_v_* vaporization temperature, *M*—molecular mass, *P_v_*—partial vapor pressure of the vapors. 

For distilled water and methanol [41], the thermal accommodation coefficient can be considered equal to unit, *κ_dW_ = κ_M_ =* 1 and for acetone is *κ_ac_ =* 0.32. The mass accommodation coefficient for vapor is quoted for *κ_vW_ =* 0.04, for acetone *κ_vac_ =* 0.026 and for methanol *κ_vM_ =* 0.056. For the vaporization in the region of liquid meniscus [42], the mass flow rate of the vaporized liquid *ṁ_v_* can be determined depending on the temperature of the liquid film, its thickness and the pressure and temperature at the vapor-liquid interface:

(33) $${\dot{m}}_{v}=a\;(T_{\delta_{fl,va}}-T_{v})+b\;(P_{l}-P_{v})\;,$$

where $T_{\delta_{fl,va}}$ is the temperature of the film of liquid from the evaporator, *T_v_*—the temperature of the liquid vapors, *P_l_, P_v_*—pressure of the liquid and of the liquid vapors, respectively. According to [42], the parameters *a* and *b* are:

(34) $$a=\frac{2\kappa_{a}}{2-\kappa_{a}}{\left(\frac{M}{2\pi RT_{\delta_{fl,va}}}\right)}^{1/2}\left(\frac{P_{v}M\;h_{\mathrm{lg}}}{RT_{v}T_{\delta_{fl,va}}}\right),$$

(35) $$b=\frac{2\kappa_{a}}{2-\kappa_{a}}{\left(\frac{M}{2\pi RT_{\delta_{fl,va}}}\right)}^{1/2}\left(\frac{V_{m,l}P_{v}}{RT_{\delta_{fl,va}}}\right)\;,$$

where *V_m,l_*—the molar volume of the liquid. The coefficient of accommodation for distilled water [41] can be considered equal to unit. The mass flow rate of the evaporated liquid in the evaporator is found applying the equation of unidirectional conduction:

(36) $${\dot{m}}_{v}=\frac{k_{eq}\left(T_{p,va}-T_{\delta_{fl,va}}\right)}{\delta_{fl,va}\;h_{\mathrm{lg}}}.$$

The temperature of the liquid from the evaporator can be calculated using the relation:

(37) $$T_{\delta_{fl,va}}=\frac{\delta_{fl,va}\;h_{\mathrm{lg}}\left[aT_{v}-b\left(P_{l}-P_{v}\right)\right]+k_{eq}T_{p,va}}{k_{eq}+a\;\delta_{fl,va}\;h_{\mathrm{lg}}}.$$

The equivalent thermal conductivity of the liquid-wick structure in the vaporization zone [43] is calculated by the relation:

(38) $$k_{eq,va}=\frac{{\dot{Q}}_{va}L_{va}}{A_{va}\left(T_{p,va}-T_{\delta_{fl,va}}\right)}.$$

**Figure 7:**
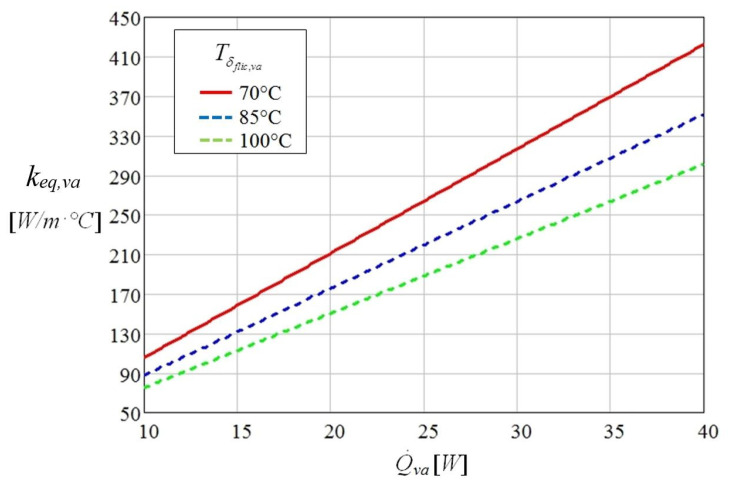
The equivalent thermal conductivity of the liquid-wick structure in the vaporization region as function of the thermal flow and temperature.

The variation of the equivalent thermal conductivity of the liquid-wick structure in the vaporization region as function of the thermal flow for different temperatures is presented in Figure 7, for the distilled water as working fluid.

The equivalent thermal conductivity of the vaporization region changes directly proportional to the applied thermal flow in the region of the evaporator and inversely proportional to the variation of temperature of the film of liquid from the microchannels of the flat MHP. From Figure 7 it is observed that for the distilled water as working liquid the equivalent thermal conductivity of the vaporization region *k_eq,va_* presents the highest increase at 70 °C and for a thermal flow of 40 W.

A source code was developed in Mathcad [2], assuming that the equivalent thermal conductivity depends on the thermal flow, the interior structure of the flat MHP, material, etc., and presents the maximum value in the region where the warm source is applied. The computations were made for the same values of the input heat ranging from 10 ÷ 40 W. The code allows for the determination of the temperature through the liquid-wick structure of a flat MHP in a transient regime.

**Figure 8:**
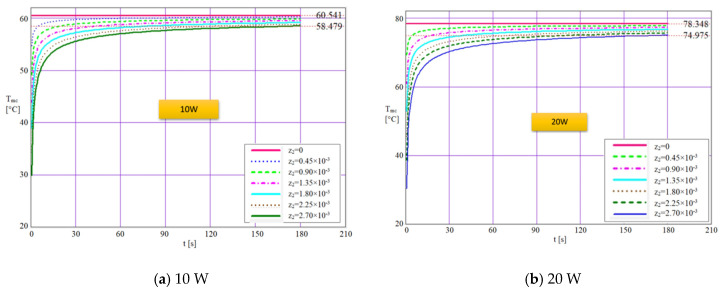

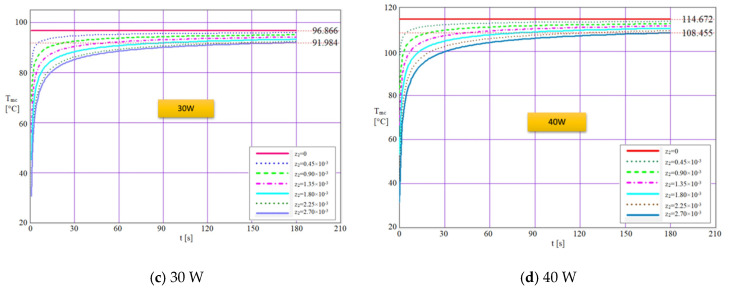
Temperature variation in time through the wall of flat MHP for different heat flow rates.

For the wall of the evaporator (Figure 2) it was adopted *δ*_1_ *+ δ*_2_ *=* 5.50 × 10^−3^ m. After the calculus was made, the plots from Figure 8a–d were traced for a time interval of 180 s (logarithmic scale), representing the variation of temperature from the base of the liquid-wick structure that is in contact with the warm source till the interior of the flat MHP. On each figure, the software shows on the upper right side the maximum values that can be reached in the structure of the flat MHP. The thickness values considered in calculus are given in the legend of each figure. The variation of temperature with time through the liquid-wick structure of flat MHP is calculated for four values of the rate of heat flow, 10, 20, 30 and 40 W for different values of the wall thickness. Theoretically, three cases can occur: the first corresponds to input heats of 10 W and 20 W when the temperature reaches a maximum of 65.410 °C and 78.348 °C, respectively, values for which the electronic components are heated but not necessarily dangerous for the adequate functioning. In this case, the flat MHP can cool the electronic device without problems, since the liquid inside it attains the vaporization temperature corresponding to the partial vacuum from inside. The second case corresponds to an input heat of 30 W when the maximum temperature reached is 96.866 °C. In this situation, the flat MHP must employ working liquids with higher vaporization temperatures capable to ensure superior heat transport capacity.

This second class of flat MHP operates at a limit if the maximum temperature of the warm source is reached. Though, it can be affirmed that a stable running regime exists. The third case refers to the input heat of 40 W when the maximum temperature reached is 114.672 °C and in this situation, the flat MHP operates under overload. For these values of input heat, the flat MHP must be designed with adequate working liquids, materials and capillary structures. The mathematical model employed demonstrates that the variation of temperature depends on the value of the input heat (that imposes the maximum temperature in the evaporation zone of the flat MHP), the physical characteristics of the material, the summated thickness of the wall and liquid-wick structure and time. The manner the rate of heat flow influences the temperature decrease is presented in Table 2.

After the transient operation, the working regime of the flat MHP becomes steady if fluctuations of the input heat are not occurring. The influence of the input heat upon the values of the temperature inside the flat MHP in the evaporation region is fundamental for their functioning both for nominal loads and overloads.

### 4.2. Modeling of Heat Transfer inside a Flat MHP

The model of heat transfer was accomplished considering both the heat and the mass transfer. The displacement of the vapors within the flat MHP from the evaporator to the condenser is subjected to a convective heat transfer. Considering $\vec{v}$—the vector of displacement of the field of heat, the equation of thermal balance for the heat transfer is:

(39) $$\frac{\partial T_{p,va}}{\partial t}+\frac{\partial }{\partial z}\left(\vec{v}T_{v}\right)=a_{p,va}\frac{\partial^{2}T_{p.va}}{\partial z^{2}}+\alpha_{co}\left(T_{v}-T_{i}\right)L_{ef}\left(y\right),$$

where *T_p,va_*—is the temperature of the wall of the evaporator, *T_v_*—temperature of vapors, *T_i_*—the temperature inside the structure of the flat MHP; *a_p,va_*—coefficient of thermal diffusivity of the evaporator’s wall, *α_co_*—the coefficient of convective transfer, *L_ef_*—the effective length of the flat MHP (see Figure 1). 

A source code was developed in MATLAB [2,44], using FTCS—a finite difference method, for numerically solving the heat transfer equation through the flat MHP, by considering *i* points in the space and *k* points in time:

(40) $$\frac{{\left(T_{i}^{k+1}-T_{i}^{k}\right)}_{p,va}}{\Delta t}+v\frac{{\left(T_{i+1}^{k+1}-T_{i-1}^{k+1}\right)}_{v}}{2\Delta z}=a_{p,va}\frac{{\left(T_{i+1}^{k+1}-2T_{i}^{k+1}+T_{i-1}^{k+1}\right)}_{p,va}}{\Delta z^{2}}+\alpha_{co}\left(T_{v}-T_{i}^{k}\right)L\left(y_{i}\right).$$

It is assumed that *v* is constant and *T_i_^k^* = *T_ini_* and Equation (40) becomes:

(41) $$\frac{{\left(T_{i}^{k+1}-T_{i}^{k}\right)}_{p,va}}{\Delta t}+v\frac{{\left(T_{i}^{k+1}-T_{i-1}^{k+1}\right)}_{v}}{2\Delta z}=a_{p,va}\frac{{\left(T_{i+1}^{k+1}-2T_{i}^{k+1}+T_{i-1}^{k+1}\right)}_{p.va}}{\Delta z^{2}}+\alpha_{co}\left(T_{v}-T_{i}^{k}\right)L_{ef}\left(y_{i}\right),$$

(42) $$\begin{array}{l} -\left(\frac{a_{p.va}\Delta t}{\Delta z^{2}}+\frac{v\Delta t}{\Delta z}\right)T_{i-1}^{k+1}+\left(2\frac{a_{p.va}\Delta t}{\Delta z^{2}}+\frac{v\Delta t}{\Delta z}+1\right)T_{i}^{k+1}-\left(\frac{a_{p.va}\Delta t}{\Delta z^{2}}\right)T_{i+1}^{k+1}=\\ =T_{i}^{k}+\alpha_{co}\left(T_{vap}-T_{i}^{k}\right)L_{ef}\left(y_{i}\right). \end{array}$$

The initialization parameters are set in the MATLAB code and for the convective transfer under the transient regime during the period of time *t* the isothermal curves are obtained. 

In order to compare the results of the model with the analytical and experimental ones, the input heat values were set in the range of 10 ÷ 40 W.

**Figure 9:**
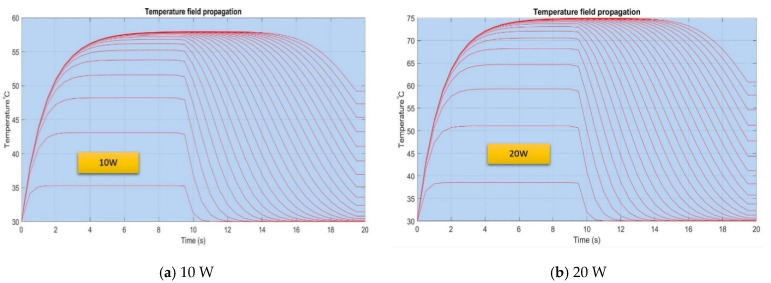

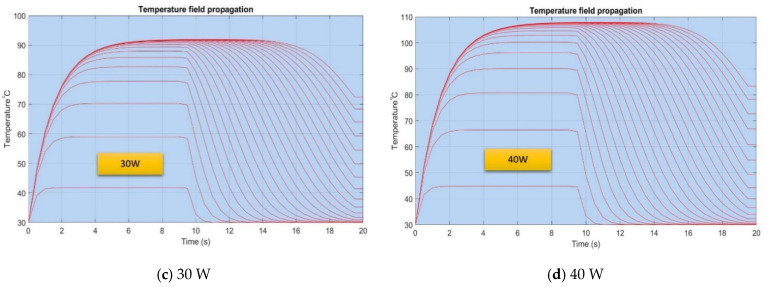
Modeling temperature variation versus time within a flat MHP.

The isothermal curves that resulted from the MATLAB simulation are represented in Figure 9 and highlight the transient evolution of the temperature for different rates of heat flows when the working liquid is distilled water. The curves are obtained for a certain number of iterations specifies in the source code. It is observed that according to Figure 9a, when the flat MHP operates at 10 W the temperature reached in the evaporation zone is only 48.7 °C compared to the maximum value attained within the structure, 58.479 °C. For the cases from Figure 9b,c, the curves present similar shapes, only the values differ in time. 

For the maximum rate of heat flow of 40 W in the case presented in Figure 9d, the temperature reached within the evaporation zone is 83.270 °C and for the internal structure, the temperature is 108.455 °C. It is noticed that the differences in temperature augment significantly as the input heat increases. The shape of the curves shows that initially, the working liquid warms during an interval of 6 ÷ 8 s until the maximum value is reached, and subsequently, the vaporization process begins.

The vaporization process is accompanied by a mass transfer process since the vapors migrate towards the condenser. This fact determines the decrease in the temperature inside the flat MHP followed by stabilization at the values indicated in the figures.

### 4.3. Result Validation

**Figure 10:**
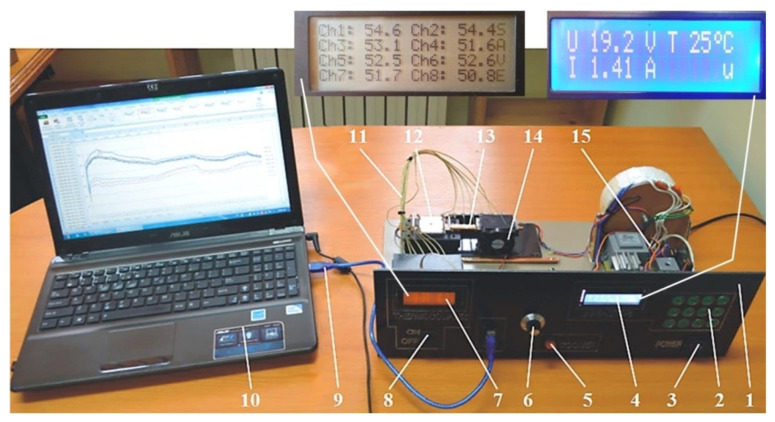
Experimental set-up for the study of heat transfer through flat MHPs [2].

In order to validate the results obtained analytically and numerically, a comparison with own experimental results as well as with other results from the literature [45,46,47], was made. The experimental data was obtained using the set-up shown in Figure 10.

The front panel of the experimental set-up 1 allows different heating regimes to be preset using the keypad 2 while switch 3 allows the set-up to be switched on and off. The parameters required to control the heating system can be monitored in real-time by means of display 4. Various heating regimes are obtained by means of a heating element 12, made by depositing a 4 Ω resistance graphite layer on a metal support with a, supplied from a current source. The set-up is fitted with a cooling control system 6 which can be switched on and off by means of switch 5. This controls a fan 14, located in the condensing area. The fan airflow can be varied by changing its operating speed. The electronic thermometer 7 equipped with eight channels, acquires data from several temperature sensors 11 and allows monitoring of the temperature along the entire length of the MHP. Switch 8 allows temperature monitoring through the flat (interchangeable) micro-thermal tubes 13. A data logger 9, linked to the electronic thermometer stores the temperatures read by the thermocouples and ensures the interface to a computer 10 via a USB port. Using data acquisition software, temperature variation graphs are obtained for eight points.

The calculus models used allow for determining the temperature difference between the hot surface and the inside of the heat pipe, in the evaporator region of the flat MHP. The used equipment allows precise evaluation of the external temperature evolution in the transient regime for the evaporator wall. In Table 3, the data obtained by the authors for the analytical experimental and FTCS modeling cases are compared with results obtained by other authors.

For the distilled water, the difference in temperature between the two faces of the liquid-wick structure is 2.06 °C for 10 W and 6.21 °C for 40 W. One can state that difference in temperature increases with the thickness of the flat MHP, so thinner walls are recommendable. But an optimum thickness must be ensured since the wall of the flat MHP must resist the pressure differences from within.

Results shown in Table 3 illustrate that the experimental data obtained by the authors are in good agreement with those determined by calculus, which means that the method of determining the temperature field within the MHP’s wall can be applied regardless of the used liquid and the heat source power. Differences found for results obtained numerically as well as for the results of other authors are due to the imposed initial conditions, used materials, different powers of the heat sources, etc. However, the curves obtained by the authors are similarly shaped to those found in the literature.

With regards to the maximum cooling capacity of MHPs, in Table 4, the values obtained by the authors are compared with those from other similar research.

By analyzing the data in Table 4 and from the practice of operating MHPs, it was deduced that there are different factors influencing the maximum heat transport capacity. It can be observed that depending on the capillary radius, used materials, working temperature and position of MHPs different functional limits can occur with a strong impact on the cooling of components.

## 5. Conclusions

Thermal tubes ensure the cooling of power electronic components by transporting the heat from the vaporization zone to the condensation zone. The investigations and results presented herein allow the formulation of the following conclusions:

1. The method, acknowledged to provide efficient vaporization and condensation, consists in creating a partial vacuum at pre-set values. The effectiveness of the cooling depends on the liquid used and on its quantity. When the working liquid is too abundant or insufficient, malfunctions of the flat thermal microtubes may occur.
2. Assessment of the maximum heat load and its influence on temperature variation of the evaporator in flat MHPs in a transient regime is useful in the field of cooling components that need fast dissipation of important heat. The present study allows determining the correlation between some parameters on the microchannel capillary radius, for three fluids and different temperatures that can be applied to different thermal control systems. Such systems can be used in various fields, such as the cooling of biological objects and drying technology.
3. In the present paper the maximum rate of heat flow that can be applied in the vaporization region of a flat MHP was found analytically as a function of the capillary radius and for different working liquids such as acetone, methanol or distilled water. Finding the maximum rate of heat flow is important for practical applications since beyond this value the functional blocking of the flat MHP, by overheating, occurs.
4. Calculations performed to determine the maximum heat transport capacity in MHPs take into account the physical characteristics of the working fluids, the geometrical parameters of the microchannels and the structure providing capillarity. The maximum heat transport capacity of MHPs is conditioned by the applied heat flux. At too high a heat flux applied in the vaporization zone, MHPs cannot be functionally stabilized if the working fluid flow rate is too low or in excess. Excess liquid flow can cause such intense vapor flow that it prevents the liquid from flowing out of the microchannels through the vaporization and promotes boundary layer detachment.
5. The capillarity limit influences the functioning of MHPs, as for low temperatures it is restricted by the reduced pressure of vapors and the high viscosity of the liquid. When temperatures increase near the critical point, latent vaporization heat, surface tension as well as maximum power, decrease drastically.
6. The decrease in the input heat takes place with the temperature increase and it is explained by the diminution of the thickness of condensation film in the vaporization region, due to the faster vaporization rate. Decreasing the rate of heat flow with increasing temperature also depends on the working fluid. The explanation for the pronounced decrease in maximum input heat for acetone and methanol is found in their much higher volatility.
7. The calculus codes developed in Mathcad permit predicting the difference in temperature between the two faces of the evaporator (exterior and interior). The hypothesis adopted is that the external temperature to the flat MHP corresponds to the warm source. The source codes consider the values of the input heat applied to the flat MHP, the material, structure and thickness of the solid walls, the working liquid, specific heat, coefficient of thermal diffusivity and the equivalent coefficient of thermal conduction, etc.
8. The selection of the working liquid must be made to ensure the cooling of the electronic device even for overloading. As known [1] the functional difference concerning a temperature disparity of over 10 °C with respect to the constructive maximum temperature may conduct to the physical collapse of the electronic components.

## Figures and Tables

**Table 1:** Thermo-physical properties of working liquids used in flat MHPs.

Working Liquid	Liquid Density	Thermal Conductivity	Surface Tension	Dynamic Viscosity	Boiling Point
Notations	*ρ_lic_*	*λ_lic_*	*σ_lic_*	*μ_lic_*	*T_sat_*
Unit	[kg/m^3^]	[W/m°C]	[N/m]	[Pa·s]	[°C]
Distilled water	996	0.632	0.0695	7.99 × 10^−4^	100
Methanol	792	0.203	0.0262	5.21 × 10^−4^	65
Acetone	791	0.181	0.0237	3.23 × 10^−4^	56

**Table 2:** The temperature drop in the walls of the flat MHP depending on the variation of the thermal flow.

Parameters	U.M.	Values			
Input rate of heat flow	[W]	10	20	30	40
Maximum temperature outside evaporator of the flat MHP	[°C]	60.541	78.348	96.866	114.672
Internal temperature inside the evaporator of the flat MHP	[°C]	58.479	74.975	91.984	108.455
Temperature difference	[°C]	2.062	3.373	4.882	6.217

**Table 3:** Comparison between present study results and the literature.

Parameters	U.M.	Values				
Heat flow	[W]	10	10	10	6	20
Thermal regime stabilization time	[s]	175	175	175	197	278
MHP surface temperature	[°C]	59.2	60.54	58.75	66.9	64.5
MHP inner wall temperature	[°C]	57.1	58.48	56.60	60.1	58.8
Temperature difference through the MHP wall	[°C]	2.1	2.06	2.15	6.8	5.7
Data source		Experimental	Analytic	Numeric	[45]	[46]

**Table 4:** MHP’s maximum heat transport capacity.

	MHP’s Heat Transport Capacity	Operating Temperature	Capillary Radius	Wall/Working Fluid
U.M.	W	°C	m	
Various functional limits (present study)	106.62	120	3.48 × 10^−4^	Cooper/distilled water
	44.43	120	3.48 × 10^−4^	Cooper/methanol
	19.12	120	3.48 × 10^−4^	Cooper/acetone
[47]—horizontal	88.70	150	6.25 × 10^−3^	Cooper/distilled water
[47]—horizontal	83.50	120	6.25 × 10^−3^	Cooper/distilled water
[47]—vertical	46.50	130	6.25 × 10^−3^	Cooper/distilled water
[47]—vertical	44.60	120	6.25 × 10^−3^	Cooper/distilled water

## Data Availability

Some or all data, models, or code generated or used during the study are available from the corresponding author by request.
